# Anticoagulation in venovenous extracorporeal membrane oxygenation

**DOI:** 10.3389/fmed.2025.1530411

**Published:** 2025-03-04

**Authors:** Carolin Jung, Thomas Stueber, Martin Mirus, Lars Heubner, Peter Markus Spieth

**Affiliations:** ^1^Department of Anesthesiology and Intensive Care Medicine, Hannover Medical School, Hannover, Germany; ^2^Department of Anesthesiology and Intensive Care Medicine, Faculty of Medicine and University Hospital Carl Gustav Carus, TUD Dresden University of Technology, Dresden, Germany

**Keywords:** ECMO, anticoagulation, personalized, ARDS, coagulopathy

## Abstract

Venovenous extracorporeal membrane oxygenation (VV-ECMO) is a lifesaving therapy in severe acute respiratory distress syndrome (ARDS). Unfortunately, bleeding and thrombotic complications occur regularly due to coagulation disorders associated with the device, the underlying disease, and the anticoagulation management. To facilitate a personalized approach to hemostasis in individuals receiving ECMO support, it is essential to assess the coagulative state of the patient while simultaneously taking into account the underlying medical condition and administered therapies.

## 1 Introduction

**Figure 1:**
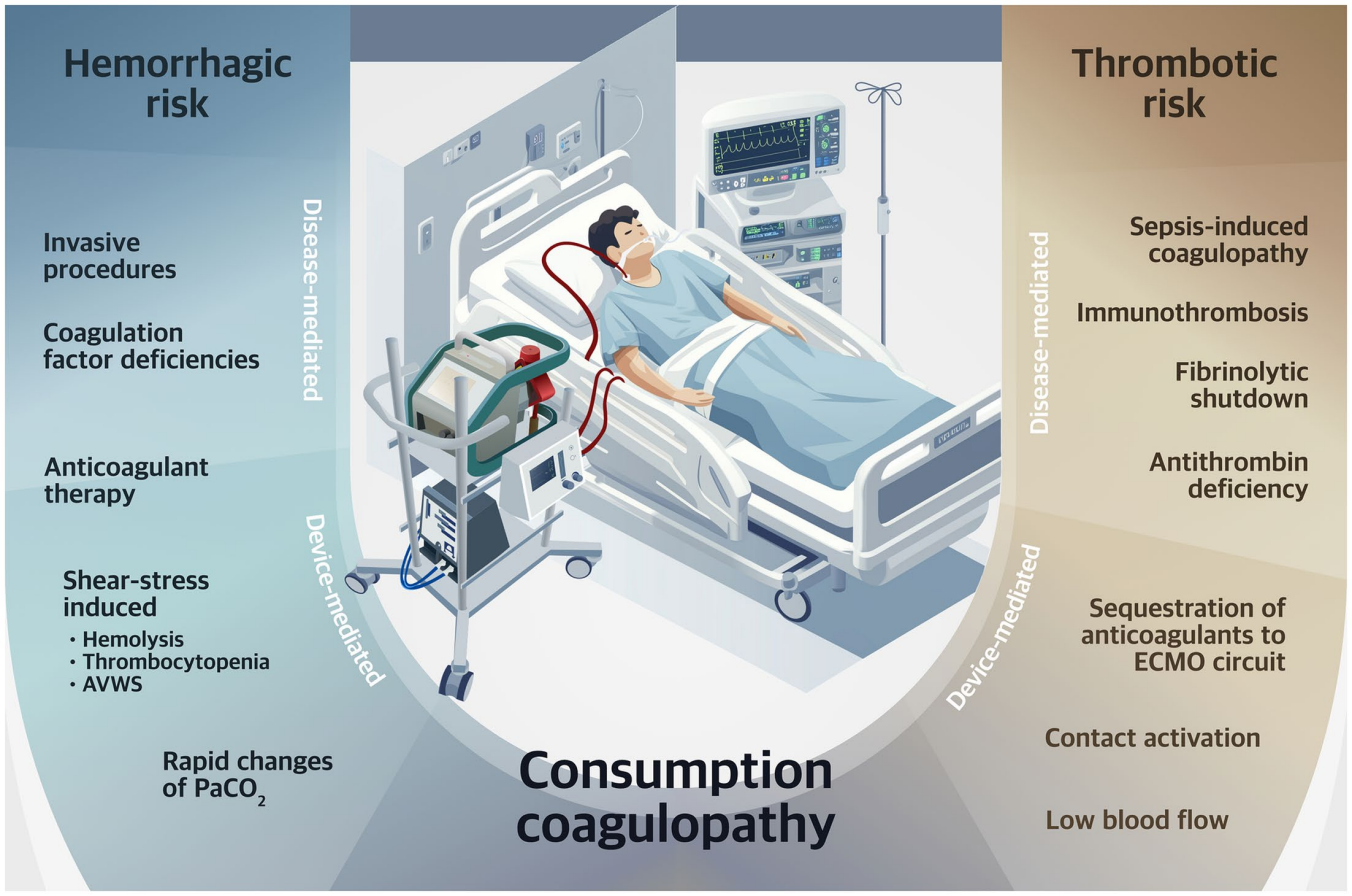
Determinants of risk for hemorrhagic and thrombotic complications during VV-ECMO. PaCO_2_, arterial carbon dioxide partial pressure; AVWS, acquired von Willebrand syndrome; VV-ECMO, venovenous extracorporeal membrane oxygenation.

**Figure 2:**
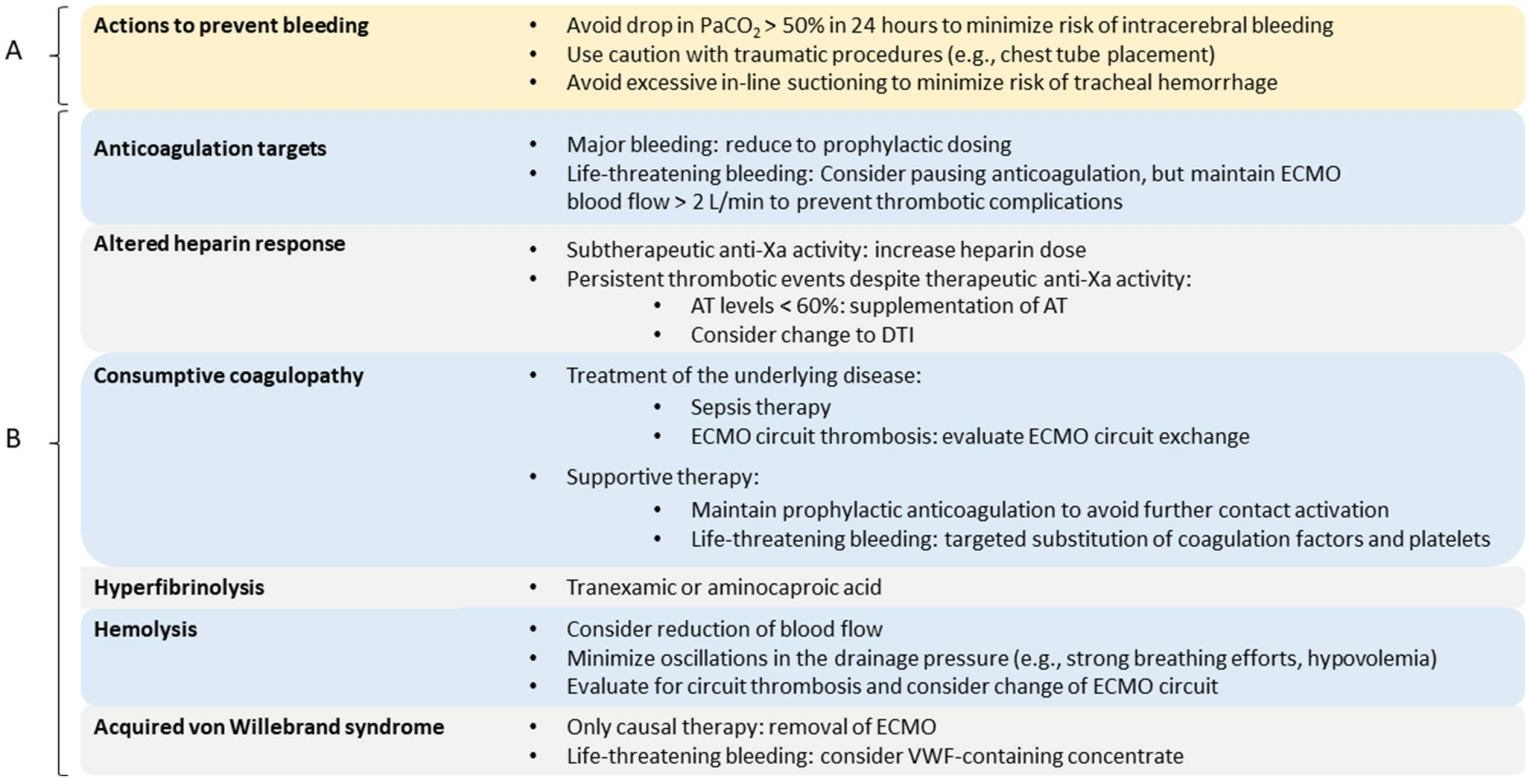
Personalized therapy. Overview of measures to prevent bleeding complications **(A)** and targeted therapeutic options in case of hemostasis disorders **(B)**. AT, antithrombin; DTI, direct thrombin inhibitor; ECMO, extracorporeal membrane oxygenation; PaCO_2_, arterial carbon dioxide partial pressure; VWF, von Willebrand factor.

Venovenous extracorporeal membrane oxygenation (VV-ECMO) can substitute the oxygenation and decarboxylation function of the lungs. VV-ECMO has demonstrated a survival advantage in patients with severe acute respiratory distress syndrome (ARDS) compared to those receiving conventional therapy (1–3). Recent guidelines recommend VV-ECMO for selected patients with severe ARDS (4, 5). While there is moderate evidence for faster resolution of organ failure in individuals receiving VV-ECMO support, there is also a markedly increased risk of bleeding and thrombotic complications (5, 6). During ECMO support, anticoagulation is necessary to prevent clot formation in the circuit and oxygenator. Thrombotic events occur more often compared to bleeding events; however, bleeding is associated with worse outcomes (7). Therefore, the risk of bleeding should be minimized while balancing the risk for thromboembolic complications. According to Extracorporeal Life Support Organization (ELSO) registry data, the incidence of thrombotic and bleeding events during ECMO support decreased during the last years (7). Bleeding or thrombotic events associated with the highest mortality were ischemic stroke and intracranial hemorrhage, followed by pulmonary and gastrointestinal bleeding (7). Critical illness, extracorporeal support and anticoagulation management influence the balance between pro- and anticoagulation (Figure 1). To apply a personalized approach to anticoagulation management in ECMO patients, a thorough understanding of the mechanisms contributing to the hemostatic disorders is necessary. This review aimed at providing a comprehensive summary of the current evidence and recommendations on anticoagulation management in VV-ECMO patients. We have structured this review to first provide an overview of thrombotic and bleeding complications in VV-ECMO (Chapter 1), then explain how the underlying disease (Chapter 2) and the extracorporeal circulation (Chapter 3) influence hemostasis, followed by the presentation of pharmacologic options for anticoagulation (Chapter 4) and its monitoring (Chapter 5). Finally, we offer a proposal for personalized coagulation management for patients with VV-ECMO (Chapter 6 and Figure 2).

### 1.1 Bleeding complications

A recent study provided detailed data on bleeding complications in a large cohort of ECMO patients (8). About 50% of patients receiving VV-ECMO support experience at least one episode of bleeding, of which 1.3% were fatal. A total of 7.7% were life-threatening bleeding complications, necessitating a packed red blood cell transfusion with or without a surgical intervention to stop the bleeding or discontinuation of ECMO (8). About two-thirds of patients experiencing bleeding complications suffer from more than one episode. After day 7, there appears to be a small but steady rise in the rate of bleeding. Most frequently, bleeding occurs at the cannulation site, followed by tracheal-pulmonary and oro-nasal bleeding. Intracranial hemorrhage is a rare complication, accounting for 3% of bleeding episodes. Bleeding from the cannulation site seems to be more common in the early stages of the ECMO course, whereas in the later stages, the incidence of bleeding from pulmonary and oro-nasal sites increases. Bleeding is associated with increased mortality, regardless of the severity of bleeding (8). Therapeutic anticoagulation is a risk factor for bleeding events in ECMO patients. As activated partial thromboplastin time (aPTT) increases, the risk of bleeding increases proportionally. In the PROTECMO study, a higher aPTT was the main determinant for the first bleeding event during VV-ECMO (8). However, the amount of anticoagulation applied does not fully explain these bleeding events, since bleeding even occurs in patients with low and no anticoagulation. Since aPTT and the dose of UFH were only weakly correlated, increases in aPTT may also be an expression of an underlying coagulopathy. An increased aPTT should therefore be considered a risk factor for progress of the underlying disease, which can lead to bleeding and thromboembolic complications, irrespective of the applied anticoagulation. There is growing evidence that a multifactorial coagulopathy develops during support with extracorporeal assist devices (9–11). This multifactorial coagulopathy seems to be fueled not only by the applied anticoagulant therapy but also by the underlying disease and interactions of the blood with the extracorporeal device (12). However, there is no standard laboratory test available that can reliably predict bleeding complications in ECMO patients.

### 1.2 Thrombotic complications

Anticoagulation management is complicated due to the fact that bleeding complications are often accompanied by concomitant thrombotic events. Thrombotic events occur in 25% of patients supported with VV-ECMO (7). The majority of data concerning thrombotic events during VV-ECMO are derived from the ELSO registry, which primarily covers circuit thrombosis, oxygenator or pump failure, and ischemic stroke (7). Whereas circuit thrombosis and oxygenator failure are frequent events, ischemic strokes are rare and account for only 1% of cases (7). Systematically collected data regarding venous thrombotic events such as cannulae-associated venous thrombosis or lung artery embolism in patients with VV-ECMO are missing. Therefore, we may currently underestimate the incidence of thrombotic events (7). During the COVID-19 pandemic, some centers reported data on the incidence of thromboembolic events on VV-ECMO. However, this information should be interpreted with caution due to the unique nature of COVID-19 related coagulopathy. Considering this, deep vein thrombosis or pulmonary embolism might occur in 35 to 51% of ECMO-patients (13, 14).

## 2 Influence of underlying disease

Changes in hemostasis caused by serious illness alter the balance between pro- and anticoagulant factors. This may lead to a prothrombotic or hemorrhagic state and significantly alters coagulation management.

### 2.1 Immunothrombosis

Severely ill patients often present with an impaired coagulation even before ECMO support (10, 15). Likely contributing factors are pathogen- and damage-associated molecular patterns, acute phase reactants, and organ failure accompanying the underlying disease. These factors may induce a prothrombotic state with successive formation of microthrombi in the microvasculature (16). When cells are damaged, as it happens during trauma, sepsis, or ARDS, molecules linked to mitochondrial damage are released into the bloodstream. The host initiates a proinflammatory response to protect from potential pathogens. In the case of a dysregulated inflammatory response, further collateral damage, especially of endothelial cells, occurs. The endothelial injury activates coagulation, resulting in the formation of microthrombi in the affected microvasculature (16). During endothelial damage, tissue factor (TF) is released into the circulation and further activates the coagulation cascade. This concept, also called “immunothrombosis,” has been established as an essential part of ARDS pathophysiology (16). Thus, a complex interplay between the immune and coagulation systems induces a systemic activation of the coagulation system. If the coagulation cascade is ongoing, exhaustion of platelets, fibrinogen and coagulation factors results in a consumption coagulopathy, which subsequently leads to bleeding complications. Furthermore, these patients often receive anticoagulants or platelet inhibitors before initiation of ECMO, which contributes to a dysfunctional baseline hemostasis (15). Hekimian et al. reported that after initiation of ECMO, markers of endogenous thrombin generation increased steadily and were accompanied by a massive increase of fibrin formation markers despite therapeutic anticoagulation (10). This was most pronounced in ARDS patients with COVID-19, which is typically associated with a procoagulant state (17). In contrast, in a small cohort of patients with VV-ECMO due to chronic obstructive pulmonary disease that had less severe inflammation, no significant changes in markers of thrombin generation or fibrin generation and degradation were seen during the first 7 days of ECMO support (18). Increased thrombin generation leads to further platelet and fibrinogen consumption, as thrombin is the strongest platelet activator. This may cause a consumption-mediated change to a hypocoagulable state with an increase of bleeding complications later in the course of ECMO support (10).

### 2.2 Impaired fibrinolytic capacity

Patients with sepsis or infectious ARDS often demonstrate a low fibrinolytic activity, that contributes to a prothrombotic state (19, 20). This cessation of fibrinolysis following the systemic activation of the fibrinolytic system is known as impaired fibrinolytic capacity. If a total resistance to fibrinolysis in whole blood viscoelastic testing occurs, this is referred to as “fibrinolytic shutdown” (21). It is most likely an acute phase response to control hemostasis that is yet incompletely understood (22).

### 2.3 Consumptive coagulopathy

Endothelial damage from severe systemic inflammation in ARDS and sepsis can lead to a consumptive coagulopathy, causing a decrease in platelets, fibrinogen, and coagulation factors, and an increase in thrombus formation, especially in the microvasculature. This results in both, an increased bleeding risk, as well as a progressive loss of organ function with increasing thrombosis. The growing thrombus formation further amplifies the coagulation cascade, therefore contributing to a vicious cycle (23). The terms “consumptive coagulopathy,” and “disseminated intravascular coagulation” (DIC) are often used synonymously. However, the diagnostic criteria for overt DIC only apply to rather advanced stages of the consumptive coagulopathy, excluding early stages of the consumptive process (24, 25).

### 2.4 Heparin resistance

The term heparin resistance is used in clinical practice if unusually high doses of heparin are required to achieve a certain therapeutic value in an anticoagulation assay. There is yet no consensus on the definition of the term “heparin resistance,” and a wide variety of thresholds defining what constitutes an adequate dose of heparin exist in the literature. Most often, a heparin dose >35,000 U/d or > 30 U/kg/h is used to discriminate an adequate from an inadequate response (26). Heparin is a substance with complicated pharmacokinetics, even in healthy individuals. The applied dose does not linearly relate to the anticoagulant effect. Heparin is eliminated by absorption in the reticuloendothelial system, cleavage in the liver, and by excretion in the urine. As a result, both liver and kidney function have an influence on heparin plasma levels (27). The elimination half-life for heparin is disproportionally longer in high doses (27). In critically ill patients, the pharmacokinetics of heparin are even more complicated. The heparin response is further dependent on a variety of factors, including the patient-specific distribution volume, alterations of anticoagulation factor levels, and neutralization of the heparin effect via nonspecific binding of heparin to various acute-phase proteins and nonendothelial surfaces, including extracorporeal circuit components (28). Independent of the applied heparin dose, aPTT is decreased by the factors VIII and fibrinogen, both being acute-phase proteins that increase in inflammatory states. This can lead to so-called “pseudo heparin resistance,” characterized by an artificially low aPTT that indicates heparin resistance, while *in vivo* a heparin-effect is present. In critically ill patients, antithrombin levels can be reduced due to decreased production or increased consumption. Heparin’s anticoagulant effect depends on its interaction with antithrombin. If antithrombin levels are very low, the heparin response may be lessened (29). All these factors contribute to significant variability in the *in vivo* response to heparin, and pose challenges in measuring its effects *in vitro* (26). An altered heparin response is common in ECMO patients, with a prevalence of 17–50% (30, 31). In patients with COVID-19, heparin resistance is even more frequent (32). It is uncertain if an altered heparin response indicates an increased thromboembolic risk. In retrospective studies, there was no association between heparin resistance and the occurrence of thromboembolic events (31).

## 3 Device-related aspects

In addition to the various changes due to the critical illness, the implantation of ECMO also affects the balance between a pro- and anticoagulant state.

### 3.1 Impact of ECMO on pharmacokinetics and -dynamics

The ECMO circuit expands the patient’s distribution volume. Furthermore, circuit components tend to sequester drugs with high protein binding and lipophilicity. This may result in higher dose requirements and should be commended with careful monitoring of essential drugs. Unfractionated heparins demonstrate a high protein binding and may therefore be affected by circuit sequestration (33).

### 3.2 ECMO-induced coagulopathy

Several factors contribute to the effect of the ECMO on hemostasis: (a) contact of the blood with artificial surfaces of the ECMO circuit; (b) impact of the already formed thrombus burden within the circuit; and (c) mechanical stress of blood cells within the circuit. To avoid thrombotic complications, anticoagulation is recommended (34, 35).

Both the contact activation pathway and the extrinsic pathway of the coagulation cascade are contributing to hemostatic changes induced by the extracorporeal circuits (36). Within minutes of contact of blood plasma with non-endothelialized surfaces, immediate adsorption of fibrinogen to the artificial surface takes place (37). This initial fibrinogen deposition on non-biological artificial material induces activation of coagulation factors of the contact activation pathway (e.g., F XI, F XII, and kallikrein-kinin system) and complement, which allows platelets and leukocytes to adhere to the non-endothelialized circuit and oxygenator surfaces (36). The coagulation cascade is further stimulated via the extrinsic tissue factor pathway. Normally, the subendothelial matrix expresses tissue factor, necessitating a vascular lesion for TF to interact with blood plasma. In certain conditions, such as sepsis-induced disseminated intravascular coagulation, TF is also expressed intravascularly on monocytes and macrophages (36). TF binds to the circulating F VII to form the TF:VIIa complex, which is a highly potent coagulation activator. Within the ECMO circuit, the oxygenator membrane has the largest surface in contact with blood and is therefore a predilection site for thrombus formation (36). There has been some effort to enhance the biocompatibility of ECMO circuits. Modern ECMO circuits come with improved coatings of the circuits and oxygenator surfaces, resulting in a significant reduction of the thrombogenic potential (38, 39). Research to improve the hemocompatibility of the extracorporeal circuit is currently ongoing.

Shear stress causes damage to blood cells and proteins passing through the ECMO pump. ECMO flow-mediated shear stress induces thrombocytopenia by shedding of the platelet receptor glycoprotein Iba (GP Iba) and subsequent increased clearance of GP Iba-depleted thrombocytes. This shear-induced thrombocytopenia develops shortly after ECMO implantation and improves rapidly after cessation of ECMO support (40). The majority of ECMO patients also show an increased level of free circulating hemoglobin, a typical marker of hemolysis (41). Risk factors for hemolysis during VV-ECMO are both very high and very low blood flows, pump head thrombosis, and the use of dual-lumen cannulae (42). While a high blood flow (> 3 L/min) seems to only minimally affect the risk of hemolysis, very low blood flows (< 2 L/min) have demonstrated a significant impact on the hemolysis risk, which is further aggravated with a flow <1 L/min (43). The low hydraulic efficiency of contemporary rotary pump systems under very low flow conditions leads to an elevated recirculation rate and, consequently, increased shear stress (43). Hemolysis induces a prothrombotic state via activation of the coagulation system and is therefore another factor contributing to thromboembolic events and consumption coagulopathy (36). Free hemoglobin further has cytotoxic properties, including vasoconstriction by nitric oxide scavenging, activation of proinflammatory cascades, and generation of oxidative stress (44, 45). Free hemoglobin has been recognized as a risk factor for acute kidney injury (46) and increased mortality in ARDS patients with VV-ECMO (47).

The growing thrombus formation initiates a vicious circle with (a) platelet activation and aggregation (primary hemostasis), (b) clotting factor activation and amplification (secondary hemostasis), and (c) consecutive consumptive coagulopathy with fibrinogen and platelet depletion (23).

### 3.3 Acquired Von Willebrand syndrome

Von Willebrand factor (VWF) is a large multimeric glycoprotein circulating in the bloodstream, that is produced by endothel cells and megakaryocyts (48). It exerts its role in primary hemostasis via mediation of platelet adhesion to the subendothelial matrix at sides of vascular damage. Additionally, it plays an important role in secondary hemostasis by preserving factor VIII levels. VWF binds to factor VIII, thus preventing its rapid elimination from the circulating blood (48). VWF comes in multimers of various sizes, whereas only large (= high molecular weight, HMW) multimers are hemostatically active (48). The absence of large VWF multimers results in a reduced platelet adhesion at sides of vascular injury, even if VWF plasma levels are normal. This qualitative deficiency in VWF activity is the pathomechanism behind the congenital Von Willebrand Disease (VWD) type 2A and typically results in an increased bleeding risk (48). VWF multimer size is regulated via the metalloproteinase ADAMTS13. Under normal circumstances, the VWF multimers are folded, hiding the binding sides for ADAMTS13. Under shear stress, VWF multimers unfold and expose the binding sides, which promotes proteolytic cleavage of these multimers via ADAMTS13 (49). Thus, in conditions of high shear stress, e.g., extracorporeal assist devices or aortic stenosis, a loss of HMW VWF multimers results in acquired von Willebrand syndrome (AVWS), which is comparable to VWD type 2A. Nearly all patients develop AVWS within 24 h after initiation of ECMO support (15, 50). It is typically fully reversible within only a few hours after ECMO explantation (50). There is a high biological plausibility that AVWS is at least contributing to ECMO-induced coagulopathy. Unfortunately, only a few studies with small sample sizes have evaluated this topic (51–55). In these small studies, no clear association of bleeding complications and loss of HMW VWF multimers became evident. Almost all patients developed AVWS, but only a subset of these patients (10–30%) developed bleeding complications (51). Currently, there is not enough evidence to conclude to what extent AVWS actually contributes to bleeding complications during ECMO support.

### 3.4 Acquired coagulation factor deficiencies

Whereas F XII deficiency is a rare condition in the general population (56), it is highly prevalent in ECMO patients (57, 58). This is most likely due to consumption of F XII via the contact activation pathway. Low F XII activity prolongs aPTT independent of heparin action, thereby limiting the validity of aPTT in monitoring UFH in these patients. Lower F XII activity was not associated with an increase in bleeding but with fewer thromboembolic complications (57). This makes F XII a potential target for future anticoagulation therapy. Furthermore, F XIII levels appear to decrease during the course of extracorporeal support (59). In small retrospective studies, decreased levels of F XIII were not only associated with bleeding, but also with a higher ICU mortality (60, 61). According to the current knowledge, F XIII stabilizes the fibrin-polymer structure and seems to enhance hemostasis without an increased risk of thromboembolic events. Therefore, F XIII supplementation may be a favorable option in critically ill patients. However, the significance of these findings has yet to be evaluated in large prospective studies.

### 3.5 Antithrombin deficiency

Antithrombin (AT) activity of less than 70% significantly raises the risk of recurrent thromboembolism in patients with a preceding deep vein thrombosis (62). The common perception that low antithrombin levels are associated with heparin resistance is challenged with the observation of the recent ATECMO study, in which antithrombin deficiency was not associated with heparin responsiveness measured via anti-Xa levels and dosing requirements. In this cohort, which included exclusively VA-ECMO patients, initiation of ECMO support was accompanied by a moderate acquired deficiency of antithrombin levels that mainly recovered within 72 h (63).

It is not clear which AT plasma level is required *in vivo* for a sufficient heparin response. In an experimental setting, the heparin response was only reduced when antithrombin plasma levels fell below 27%. Even in plasma completely depleted of AT, prolongation of aPTT to therapeutic levels could be achieved with an increased dosage (64). A similar observation was made with the plasma of medical ICU patients, in which the greatest effect of *in vitro* AT supplementation was seen only when antithrombin levels dropped below 40% (29). AT levels as low as <40% were seen in less than 5% of these patients (29).

In a small RCT, AT substitution to maintain a plasma level of 80–120% did not reduce heparin requirement and had no beneficial effect on the occurrence of bleeding or thrombotic complications (65). Furthermore, AT supplementation had no effect on anti-Xa activity, indicating that supplementing AT to normal levels did not increase heparin-antithrombin activity. In both groups, there was only a modest reduction in AT levels during the observation period, with a median AT level of 84% (IQR 69–98%) in the control group (65). A subgroup analysis of this study demonstrated a significant effect on heparin dosing requirement only when AT levels were less than 60% (65). A recent meta-analysis of RCTs found no benefit of AT substitution in septic patients, while the risk of bleeding complications was increased in the group treated with AT (66). On the other hand, a subgroup analysis of the KyberSept trial demonstrated a positive impact on 90-day survival in the intention-to-treat analysis in patients with confirmed DIC and AT substitution (67). Furthermore, a recently published retrospective study from Japan showed that higher AT-III values compared to baseline were associated with better 28-day survival, with an 80% cut-off for post-treatment AT-III activity as a favorable target in DIC patients (68).

Even though the evidence is currently inconclusive, guidelines make suggestions regarding antithrombin substitution (34, 69) and therefore routine monitoring of antithrombin activity and substitution of antithrombin deficiencies can be considered. However, the optimal target is still under debate and further research is required.

## 4 Anticoagulants

Although anticoagulation is commonly used during ECMO, there is considerable research focusing on limiting anticoagulation. Currently, unfractionated heparin (UFH) is the standard anticoagulant for ECMO in most centers. A recent observational study reports its application in approximately 75% of ECMO days, followed by bivalirudin in 5% (8). Nevertheless, changes in the anticoagulation strategy during an ECMO course are common (8), highlighting the complexity of hemostasis management in these patients. While the Scientific and Standardization Committee on Perioperative and Critical Care Haemostasis and Thrombosis of the International Society on Thrombosis and Haemostasis (ISTH) recommends UFH as the primary anticoagulant of choice for ECMO support (35), the Extracorporeal Life Support Organization (ELSO) 2021 Adult and Pediatric Anticoagulation Guideline avoids recommending a specific anticoagulant due to the paucity of evidence (34).

### 4.1 Unfractionated heparin

UFH exerts its anticoagulant effect by binding and enhancing the activity of antithrombin. As a result, heparin’s clinical efficacy is dependent on AT. The heparin-antithrombin complex inactivates several procoagulant enzymes, including thrombin (factor IIa), activated factor Xa, and activated factors XIa and XIIa (27). Inactivation of thrombin inhibits platelet aggregation and fibrin formation (70). Furthermore, through its interactions with endothelial cells and plasma proteins, heparin has pleiotropic effects on hemostasis, resulting in a variable anticoagulant response (70). The advantages of using UFH for anticoagulation include its affordable therapeutic costs, the availability of an antidot, and the extensive experience in managing anticoagulation with this ancient substance. However, patients with elevated acute-phase reactants may have altered heparin responses, complicating drug titration in this patient cohort. Heparins can further trigger heparin-induced thrombocytopenia type II (HIT II) as a severe complication. Reports indicate a markedly higher incidence of HIT II in patients receiving ECMO, ranging from 3.5 to 4.5% (8, 71), compared to the general population of patients exposed to UFH for medical and surgical indications, in which HIT II occurs in 0.5–1% of cases (72).

### 4.2 Low-molecular-weight heparin during ECMO

LMWH are heparin-derivatives with a reduced ability to bind to plasma proteins and cells due to their smaller size. This results in more predictable pharmacokinetics, a longer plasma half-life, and reduced risk of adverse effects such as heparin-induced thrombocytopenia (73). LMWHs are usually administered subcutaneously in weight-adjusted doses once to twice daily, which makes handling of these substances comfortable. However, patients with renal insufficiency are at risk of accumulation (73). Furthermore, the absorption of substances applied subcutaneously may be unpredictable in critically ill patients (74). In patients with shock and vasopressors, redistribution of blood flow to the central organs reduces skin perfusion. Intravenous enoxaparin has shown similar efficiency to unfractionated heparin, accompanied by a favorable safety profile when administered as a bolus during percutaneous coronary procedures (75). In the future, the application of LMWH intravenously may also be an option in critically ill patients (76). Unfortunately, there is currently limited evidence regarding the safety of LMWH for ECMO, particularly if applied intravenously. Mostly retrospective observational data suggest that anticoagulation management with a therapeutic dose of LMWH (77, 78) or a prophylactic dose of LMWH (79, 80) may be sufficient to prevent thromboembolic events without increasing adverse events. Still, the currently available evidence is not sufficient to give a recommendation regarding the use of LMWH in ECMO. However, there are currently several studies that will likely shed light on this topic. The multicentric randomized RATE trial (NCT04536272) is a three-armed study, comparing a higher vs. a lower anticoagulation target with UFH vs. LMWH in a therapeutic dose in patients with VA- or VV-ECMO. The primary outcomes will be thrombotic or bleeding complications, as well as mortality. The A-FREE ECMO trial (NCT04273607) will examine the safety and feasibility of VV-ECMO with prophylactic instead of therapeutic anticoagulation. The outcome will include ECMO-associated thrombotic complications, bleeding complications, and coagulation parameters during the ECMO run.

### 4.3 Direct thrombin inhibitors

Direct thrombin inhibitors (DTI) are an alternative to anticoagulation with heparins that are increasingly used in patients supported with extracorporeal assist devices. DTI bind directly, selectively, and reversibly to the active site of thrombin. They do not attach to other plasma proteins like heparins do, which promises a more predictable response. Whereas the effect of heparin is dependent on the availability of AT, DTI are not. In contrast to heparins, which inactivate mostly soluble but not fibrin-bound thrombin, DTI show greater efficacy by inactivating both soluble thrombin as well as thrombin bound to fibrin or fibrin degradation products. An advantage of DTI over heparins is that they do not cause heparin-induced thrombocytopenia (HIT) (81). Typical indications for DTI are HIT and percutaneous coronary interventions. They are also used off-label in cases of heparin resistance (82), during ECMO support (83), and in severely prothrombotic states such as sepsis (84). Whereas argatroban is more widely used in Europe, bivalirudin is more common in Northern America (8). The drawbacks of DTI include a lack of experience in managing these substances, monitoring, and the absence of a reversal agent when necessary. Furthermore, monitoring of these substances is more complex due to the limited availability of specific laboratory testing (35). In a current multicentric observational study, a trend toward increased bleeding complications was observed in patients receiving direct thrombin inhibitors for VV-ECMO (8), which may have been mediated by the challenges in monitoring these substances.

#### 4.3.1 Argatroban

Argatroban provides a reversible inhibition of thrombin. It is predominantly metabolized hepatically and has an elimination half-life of 30 to 50 min. In patients with hepatic impairment, dose adjustment is necessary. No adjustment is required in patients with renal insufficiency (35). Currently, there is insufficient evidence to support argatroban instead of UFH for anticoagulation during ECMO support if there are no contraindications for UFH. There are yet only small retrospective studies comparing the safety and efficacy of these two anticoagulation regimes in non-HIT patients (83, 85, 86). No differences regarding bleeding or thrombotic complications were observed between groups in these studies. Despite suggested in the summary of product characteristics, the use of aPTT for monitoring has proven unsuitable and the recommended dosages would lead to overdosing in critically ill patients (87). In critically ill patients, a reduction of the initial infusion rate to 0.2–0.5 μg/kg/min is recommended. During the up-titration phase, we recommend close monitoring with a specific drug monitoring (e.g., diluted thrombin time (dTT) or a chromogenic anti-IIa assay) every 2 to 4 h. Once a steady state has been established, the monitoring interval can be reduced to once daily.

#### 4.3.2 Bivalirudin

Bivalirudin also provides its inhibitory effect via reversible direct binding to thrombin. It has an elimination half-time of 25 min. Bivalirudin is eliminated primarily through proteolytic cleavage, with 20% also being cleared in the kidneys (88). Therefore, dose adjustments are necessary in patients with reduced kidney function (35). There is little evidence regarding the use of bivalirudin in ECMO patients. There are currently mostly retrospective studies comparing bivalirudin to UFH for anticoagulation in this patient cohort (89, 90). In these retrospective case–control studies, the application of bivalirudin instead of UFH seemed to be safe and feasible (89, 90). It also appeared to be associated with a reduced rate of patient- and in-circuit-thrombosis (89), bleeding complications (90), as well as a reduced mortality (89), but these findings were inconsistent. The evaluation of the safety and efficacy of an anticoagulation strategy with bivalirudin instead of UFH in patients without HIT or heparin resistance in large prospective randomized controlled trials is still due (NCT05959252). Until then, anticoagulation with bivalirudin remains a viable option for ECMO patients if contraindications to UFH are present. As an initial dose, bivalirudin is usually started with an infusion rate of 0.02–0.05 μg/kg/min in ECMO patients (35).

## 5 Anticoagulation strategies

### 5.1 Anticoagulation targets

Current guidelines still recommend therapeutic anticoagulation targets (35). The ISTH suggests aiming for an anti-Xa level of 0.3–0.5 IU/mL, an aPTT of 50–70s or 2–2.5 times above the normal level (35). This approach should be questioned, as these recommendations have hardly been adapted to the described developments of new, less coagulation-activating circuits. There is currently a discussion regarding lower anticoagulation targets for ECMO, as the mortality associated with bleeding complications surpasses that of thrombotic events (7, 8, 91). Unfortunately, there is currently insufficient evidence considering different dosing regimens of heparin. Observational data and small pilot RCTs showed lower bleeding complications without increasing the incidence of thromboembolic events (92–94) with lower anticoagulation targets. Notably, the anticoagulation target in the EOLIA study was 40–55 s or anti-Xa 0.2–0.3 IU/mL (6), which is lower than is currently recommended. This aligns with data from the recent multicentric PROTECMO study, in which the average aPTT was 40–60s in the participating centers (8). Retrospective data indicate a lower bleeding incidence, with comparable thromboembolic events in lower versus higher anticoagulation targets (95).

### 5.2 Avoiding anticoagulation

Running ECMO without anticoagulation is widely applied in clinical practice, particularly in cases of hemorrhage. According to a recent multicentric international observational study, about 20% of ECMO therapies were initiated without any anticoagulation. Initiating ECMO without any anticoagulation was the second most performed anticoagulation procedure beside UFH. Running ECMO without any anticoagulation was maintained for approximately 50% of days (8). Individuals receiving no anticoagulation differed regarding their baseline variables. They had more often a non-infectious cause of ARDS, such as trauma, burns, or graft failure after lung transplantation. One third of these individuals had surgery in the last 7 days prior to initiation of ECMO, which was significantly more frequent than in individuals receiving anticoagulants for initiation of ECMO support (8). Although it is a frequently adopted procedure, data regarding the safety of this procedure are currently based mainly on retrospective data (96, 97). In these studies, no significant differences regarding bleeding or thrombotic complications have been reported (96, 97). In a recent retrospective study in traumatic patients with VV-ECMO, patients receiving no anticoagulation had a lower thrombocyte count, more transfusions requirements and more associated hemorrhage in CT imaging. Since the patients in the no anticoagulation cohort were more severely ill and had a significantly higher baseline aPTT level, these differences were most likely explained with confounding by baseline severity of illness (97). Interpretation of study results regarding this topic is further complicated by a variable use of the term “no anticoagulation.” Almost half of the patients included in a meta-analysis regarding the safety of ECMO without anticoagulation received other anticoagulants or heparin in a prophylactic dose (96).

From a pathophysiological perspective, appropriate caution is called for when running ECMO without any anticoagulation. The circuit’s heparin coating partially accounts for the risk of thromboembolic events. However, the membrane oxygenator’s surface is not heparin-coated, despite being the primary surface of contact activation. To avoid aggravation of consumptive coagulopathy via the contact activation pathway, continuing anticoagulation at least in a prophylactic dose seems advisable (23). Since low blood flow rates are linked to an increased risk of hemolysis and thrombotic complications, maintaining an adequately high blood flow (> 2.5–3 L/min) is recommended during anticoagulation free ECMO (98). Currently, the ISTH advises against the routine use of no anticoagulation during ECMO support (35). Pharmacological inhibition of factors involved in the contact pathway (F XI/XII) may have the potential to optimize anticoagulation management in ECMO patients in the future since they may prevent contact pathway-induced thrombosis without elevating the risk of bleeding (99).

### 5.3 Monitoring strategies

The monitoring strategy depends on the choice of anticoagulant drug as well as the availability of the corresponding tests. Currently, the search for the optimal strategy to monitor UFH, LMWH, and DTI is ongoing. Given the potential pharmacokinetic interactions between the ECMO circuit and administered medications, a specific drug monitoring of essential drugs is warranted to avoid underdosing.

#### 5.3.1 Monitoring of anticoagulation with unfractionated heparin

Tests for monitoring of UFH include:

- Anti-Xa assay.
- Activated partial thrombin time (aPTT).
- Viscoelastic Hemostatic Assay (VHA).

Anticoagulation with UFH can be monitored with global tests that measure time to clot formation (aPTT, ACT, or viscoelastic hemostatic assays) and direct measurements of anti-Xa inhibition through heparin (anti-Xa assay). The most commonly used test to monitor anticoagulation with UFH is aPTT (8). However, interpreting global coagulation tests, such as aPTT, in critically ill patients is complex. Global coagulation tests are affected by several factors, including acquired coagulation factor deficiencies (factor XI and antithrombin), platelet function, acute phase proteins (factor VIII, fibrinogen), and hemodilution (100–102). In critically ill patients, baseline aPTT frequently diverges from that of normal controls. High amounts of acute phase reactants, like fibrinogen and factor VIII, may lead to a reduced aPTT, obscuring the impact of administered UFH (34).

In ECMO patients, there is a considerable difference between the various assays, whereas anti-Xa assays provide the best correlation to the applied heparin doses (103, 104). According to a meta-analysis of observational studies, anti-Xa guided anticoagulation is linked to fewer bleeding complications and lower mortality without an increase in the rate of thromboembolic events when compared with time-based anticoagulation monitoring (105). The ISTH suggests using anti-Xa assays with a target of 0.3–0.5 U/mL to monitor anticoagulation with UFH for patients receiving ECMO support (35). Even in cases of antithrombin deficiency, anti-Xa assays demonstrate good clinical adequacy regarding patient heparin levels (29). The analytical accuracy of chromogenic assays, such as the anti-Xa assay, is compromised in all states of pigmented or opaque plasma. An anti-Xa assay may therefore underestimate the UFH effect in hyperbilirubinemia, severe hemolysis, or hypertriglyceridemia (34). However, aPTT is also influenced by hemolysis. Elevated free hemoglobin reduces aPTT and anti-Xa activity; increased bilirubin prolongs aPTT and decreases anti-Xa activity (106). Some laboratories add exogenous antithrombin-III to the anti-Xa assay. If such an antithrombin III-independent assay is used, the *in vivo* effect of UFH may be overestimated when patient antithrombin III levels are reduced (34, 107). However, the effect of AT supplementation on the diagnostic accuracy of the heparin level estimation appears to be small and may only be relevant for severely (< 40%) reduced AT levels. In an *in vitro* study, there was a high agreement in patient classification between assays with and without exogenous AT supplementation (29). While anti-Xa is the more accurate measure of the heparin effect, time-based coagulation assays like aPTT provide complimentary information regarding co-existent hemostatic disorders (34, 103). Given the limitations of each test in estimating the *in vivo* heparin effect, it seems reasonable to not rely on any single assay. Combining these tests gives a more comprehensive picture of the hemostatic state of these complex patients.

ACT has been used for decades during procedures that require a very high level of heparinization, e.g., during cardiopulmonary bypass in cardiac surgery or interventional coronary angioplasty. Due to its limited informative value regarding the response to heparin in therapeutic and prophylactic doses, monitoring anticoagulation in ECMO patients with ACT is increasingly considered historical (34). Regardless of that, ACT monitoring is still the second most frequent applied coagulation test after aPTT (8). Considering these facts, we believe ACT should not be used for monitoring in this context.

#### 5.3.2 Monitoring of anticoagulation therapy with direct thrombin inhibitors

Tests for monitoring of DTI include:

- Anti-IIa assays: dTT, ECA.
- VHA

Like with UFH, the ISTH recommends routine monitoring of DTI using aPTT measurements, targeting an elevation of 2–2.5 above the normal level (35). Similar to UFH, there are concerns regarding the validity of aPTT measurements in this context. aPTT is influenced by coagulation factor deficiencies (e.g., factors VIII, IX, XI, and XII), hypercoagulant states, and the presence of lupus anticoagulants in antiphospholipid syndrome (108). These cofactors, which are highly prevalent in critically ill patients, make aPTT measurements difficult to interpret in these patients and may result in incorrect dosing of these substances. Indeed, aPTT shows a poor correlation to DTI levels in critically ill patients (87, 109–111). A major problem of aPTT monitoring of DTI is that aPTT measurements are in particular insensitive to high doses of these drugs. For argatroban, a nonlinear relationship with a flattening of the dose response over a concentration of 1 μg/mL has been demonstrated (110, 112). From clinical experience, the aPTT is not suitable for the safe management of argatroban therapy in critically ill patients. We recently demonstrated that aPTT measurements failed to detect argatroban overdosing in a cohort of critically ill patients. In the majority of cases in which argatroban plasma levels were in the supratherapeutic range, aPTT suggested subtherapeutic values. This would misleadingly have implied increasing the argatroban dose if only aPTT measurements would have been available (87). Considering the outlined concerns regarding aPTT for monitoring of DTI, the addition of more specific drug monitoring seems advisable, especially in cases of abnormal baseline aPTT, which may be reflective of an underlying hemostatic disorder complicating aPTT interpretation, or if there is no response in aPTT to dose escalation. It should also be taken into account when bleeding or thrombotic complications occur. If available, the ISTH suggests using a calibrated anti-IIa assay for specific monitoring of DTI (35).

Anti-IIa assays quantify thrombin (F IIa) inhibition. These assays are either chromogenic assays or clot-based methods. Some of these assays utilize ecarin, which is extracted from a viper venom, to induce activation of prothrombin to meizothrombin. A theoretical advantage of these ecarin-based assays is their greater specificity to direct thrombin inhibitors, since heparin exerts no inhibitory effect on meizothrombin (113). Common anti-IIa assays are diluted thrombin time (dTT), ecarin clotting time (ECT), and ecarin chromogenic assay (ECA), whereas dTT and ECT are clot-based assays. Since anti-IIa assays show a proportional relationship to plasma concentrations of argatroban and bivalirudin, they can be used to estimate the plasma levels after calibration to the respective drug (113). Anti-IIa assays are not affected by deficiency of vitamin k dependent coagulation factors, hypercoagulant states, or the availability of lupus anticoagulants (108). Unfortunately, anti-IIa assays are still considered to be research assays with limited availability in most centers (34).

Although not formally licensed for monitoring of anticoagulation with DTI (34), all DTI induce a prolongation of prothrombin time, commonly reported as the International Normalized Ratio (INR). Argatroban is the DTI with the most pronounced effect on INR (114).

#### 5.3.3 Functional testing of hemostasis

With VHA, assessment of clot initiation, clot strength and stability, and the presence of hyper- or hypofibrinolysis is possible. Typically performed with whole blood, VHA offer dynamic, real-time evaluations of clot formation and dissolution, reflecting the contributions of cellular components and coagulation factors. Key parameters measured by VHAs include: Clotting Time (CT) or Reaction Time (R): These parameters represent the latency period from the initiation of the test to the onset of clot formation. Prolongation of CT or R during ECMO support may indicate deficiencies in coagulation factors or the presence of anticoagulants like heparin. Monitoring these parameters can aid in adjusting anticoagulant dosing to maintain optimal hemostasis (115). While VHA have not yet been validated to predict bleeding or thrombotic complications specifically in ECMO patients, it is commonly used to guide the administration of hemostatic therapies (34). Current evidence regarding the prediction of bleeding or thrombotic complications is still inconclusive (59, 116–118). However, VHA is increasingly utilized in cases of unexplained bleeding and may assist in guiding anticoagulation therapy in various clinical contexts (119). In bleeding patients, combining standard coagulation tests with VHA may produce a more comprehensive picture of the hemostasis of the individual ECMO patient. Integrating VHAs with anti-Xa activity measurements enhances anticoagulation management. While anti-Xa assays provide quantitative data on heparin levels, VHAs offer qualitative insights into the overall coagulation process, including coagulation factor deficiency and fibrinolysis. This combination allows for a more tailored approach to anticoagulation therapy, potentially reducing the risk of bleeding or thrombotic complications (120). Additionally, VHA may detect overdosing of UFH or DTI with high sensitivity and without delay, particularly when specific tests, such as anti-Xa for UFH or dTT for DTI, are unavailable. Furthermore, VHA can identify hyperfibrinolysis or hypofibrinolysis using specific test agents (e.g., the TPA-test in ClotPro®), which is not reflected by standard coagulation monitoring. Furthermore, viscoelastic testing is readily available at the bedside and facilitates rapid decision-making, offering a significant advantage in critical situations where standard laboratory testing are too slow.

### 5.4 Diagnostic approach in bleeding patients

Because mild bleeding is often the precursor of more severe bleeding complications (8), it should always prompt a more thorough assessment of the patient’s hemostatic state. If the underlying condition for the acquired coagulopathy is not addressed in a timely manner, aggravation of the coagulopathy may occur via consumption of coagulant factors and platelets. Table 1 provides a personalized diagnostic approach for evaluating hemostasis in VV-ECMO patients. All patients should undergo standardized routine laboratory testing (Table 1) at least once daily, which covers a broad picture of the hemostasis, anticoagulant drug monitoring, as well as risk markers for emerging complications such as hemolysis and consumption coagulopathy. A thorough clinical examination of the patient, visual inspection for thrombus formation within the ECMO circuit, as well as an evaluation of ECMO pressures and gas exchange capabilities, should complement the routine diagnostic testing. When complications emerge, an extended diagnostic assessment that accounts for patient-specific risk factors should be conducted (Table 2).

**Table 1:** Standard laboratory screening for hemostatic disorders during ECMO.

Parameter	When to perform	Screening for
Hemoglobin, hematocrit	Thrice daily*	Blood loss
Specific anticoagulation assay (anti-Xa, anti-IIa)	Twice daily	Anticoagulation monitoring
Platelets	Once daily	HIT-II, consumption
aPTT, INR	Once daily	Coagulopathy
Plasma free hemoglobin	Once daily	Hemolysis
Fibrinogen	Once daily	Consumption
D-dimer	Once daily	Consumption

*If hemoglobin and hematocrit are also given in the blood gas analyses, a once-daily sample is sufficient. Adapted from Mirus et al. (12).

**Table 2:** Adaptive diagnostic approach based on specific patient characteristics.

Clinical suspicion of…	Parameter
Unspecific bleeding	Viscoelastic hemostasis assay Clotting factors V, XIII VWF: Act/Ag ratio or VWF:CB/Ag ratio
Circuit thrombosis	D-dimer Visual inspection: clot formation in the ECMO circuit/oxygenator? Increase in ECMO transmembrane pressures? Decrease in ECMO gas exchange capabilities? Fibrinolytic capacity
Hemolysis	Plasma free hemoglobin >0,5 g/L Fragmentocytes Haptoglobin Lactate dehydrogenase Indirect bilirubin Visual inspection: hemoglobinuria?
Sepsis-induced coagulopathy	Leukocytes C-reactive protein Procalcitonin IL-6 Blood cultures Clinical inspection: increase in SOFA score?
Heparin resistance	Antithrombin Anti-Xa assay
Heparin-induced thrombocytopenia	4 T score PF4 ELISA If positive: Heparin-induced platelet activation (HIPA)-test or Serotonin release assay (SRA) for confirmation of HIT
Consumptive coagulopathy	D-dimer Fibrinogen Clotting factors Platelets Fibrinolytic capacity Viscoelastic hemostasis assay Clinical evaluation: potential trigger for consumptive coagulopathy?

#### 5.4.1 Consumptive coagulopathy

Consumptive coagulopathy is characterized by a depletion of coagulation factors (fibrinogen, platelets, and clotting factors); elevated D-dimer indicating thrombus formation in the microvasculature or on foreign surfaces; often increased aPTT and INR, but no effect on specific drug monitoring tests like the anti-Xa assay; and a tendency to bleed (23). Consumptive coagulopathy should trigger the search for the underlying condition, which may, for instance, be sepsis, hemolysis, or circuit thrombosis.

#### 5.4.2 Circuit-thrombosis

Suspicion of a relevant oxygenator thrombosis should occur if there is a constant increase in transmembrane pressures despite a stable blood flow rate, and when the gas transfer efficiency of the oxygenator is reduced (98). Severe hemolysis during ECMO may occur secondary to pump thrombosis (121). Hemolysis is indicated by an increase of plasma free hemoglobin to more than 0.5 g/L and the consumption of haptoglobin. Since free hemoglobin may also be elevated in other conditions, such as sepsis, additional laboratory markers, including haptoglobin, lactate dehydrogenase, indirect bilirubin and erythrocyte fragmentation should be obtained to secure the diagnosis (98). Surrogate parameters of consumption coagulopathy, such as high D-dimer, low fibrinogen, and low platelets, are often present during clinically relevant circuit thrombosis.

#### 5.4.3 Thrombocytopenia

Thrombocytopenia is very common in ECMO. Patients receiving heparins that develop thrombocytopenia and have an intermediate risk for HIT according to the 4 T score (122) should undergo testing for heparin-induced thrombocytopenia. Thrombocytopenia is frequently observed in critically ill patients and can be caused by several factors, ranging from pseudo-thrombocytopenia as a laboratory artifact caused by platelet aggregation to severe arterial embolisms caused by HIT or DIC. Correct diagnostics is quite challenging and often requires an interdisciplinary diagnostic approach and support by experts in laboratory medicine, hematology, or hemostaseology. A low platelet count might be caused by shear-stress-induced shedding of thrombocytes, sepsis-induced coagulopathy, DIC, immune thrombocytopenia, drug-induced thrombocytopenia, thrombotic thrombocytopenic purpura, thrombocytopenia caused by GP-IIb/IIIa platelet inhibitors, post-transfusion purpura, or antiphospholipid syndrome.

#### 5.4.4 Acquired Von Willebrand syndrome

Diagnostics of device-induced AVWS is challenging. Routine coagulation tests do not detect AVWS. Since the pathomechanism of ECMO-induced AVWS involves the loss of HMW VWF multimers, standard testing for quantitative and qualitative VWF deficiencies (VWF activity assay (VWF:Act), VWF antigen assay (VWF:Ag), and VWF/collagen binding assay (VWF:CB)) may not be able to sufficiently reflect the impaired VWF function. Studies evaluating AVWS have used HMW VWF multimer assays and/or a VWF:Act/Ag ratio with a cut-off of <0,7 or a VWF:CB/Ag ratio with a cut-off of <0,7 as a surrogate parameter for a loss of large VWF multimers (50). The current standard for laboratory diagnosis of AVWS is the analysis of specific VWF high-molecular-weight multimers through optical density measurement.

## 6 Personalized therapy

### 6.1 Reducing anticoagulation

Minor episodes of bleeding, for instance at the cannulae insertion sites, are frequent. Such episodes should prompt the evaluation of a potential reversible underlying cause, but they frequently do not require an alteration of the anticoagulation regime. When major bleeding complications occur during therapeutic anticoagulation, anticoagulation targets should be reduced to prophylactic levels, if not already applied. Life-threatening bleeding complications may even necessitate the complete suspension of anticoagulation. Pausing anticoagulation during ECMO support is possible even for longer periods. During paused anticoagulation, it is recommended to keep an ECMO blood flow >2 L/min to keep the risk of thrombotic complications low (98). In order to avoid further aggravation of the coagulopathy via consumption coagulopathy triggered by the ECMO surfaces, at least a prophylactic dose of anticoagulation (for example, 200–300 U UFH/h) should be kept if possible. It is further recommended to closely monitor coagulation parameters (Table 2) as well as transmembrane pressures and gas exchange of the ECMO during anticoagulation-free runs for early detection of consumption coagulopathy (23).

### 6.2 Managing altered heparin response

If suspicion of an altered heparin response arises based on aPTT measurements, daily monitoring should be extended with a chromogenic anti-Xa assay. In patients with a subtherapeutic anti-Xa activity, heparin dosing can be escalated. If there are persistent thromboembolic events such as recurrent ECMO oxygenator clotting despite therapeutic anti-Xa activity, evaluation of antithrombin level and supplementation of anti-thrombin in cases of AT <60% may be reasonable. Alternatively, a switch to a direct thrombin inhibitor can be evaluated (100).

### 6.3 Consumptive coagulopathy

Treatment of a consumptive coagulopathy with transfusion of platelets or frozen plasma concentrate and substitution of coagulation factors may aggravate thrombus formation and therefore feed the vicious circle with a subsequently increased bleeding tendency. The only causal therapy is to treat the underlying disease, e.g., focus control and antibiotic treatment in sepsis-induced DIC. If ECMO support is further required, a change of the circuit may decrease the already formed thrombus burden and therefore mitigate the consumption coagulopathy. Observational studies have shown that a significant thrombosis of the ECMO circuit that results in a loss of function is preceded by a rise of d-dimer and a fall of fibrinogen and platelets, indicating the presence of a consumptive coagulopathy (9, 123, 124). Exchange of ECMO circuits with significant clot formation can result in a recovery of platelets and fibrinogen (123, 124). In a recent study, an ECMO circuit exchange in patients with severe and persistent bleeding decreased clinical bleedings and transfusion requirements (123). To prevent further contact activation, anticoagulation at least in a prophylactic dose may be advisable in patients with consumptive coagulopathy (23). During life-threatening bleeding complications, a targeted substitution of coagulation factors and platelets will be required. However, the correct diagnosis of the underlying disease is essential for adequate treatment. Bedside whole blood viscoelastic testing might help understand the impact on global hemostasis, and the assessment of fibrinolytic capacity is crucial in these patients.

### 6.4 Hyperfibrinolysis

Antifibrinolytics are only effective when there is actual hyperfibrinolysis (22). Even in bleeding patients, hyperfibrinolysis is rare due to the high incidence of fibrinolytic shutdown in ARDS patients (15, 117). If VHA reveals hyperfibrinolysis in bleeding patients, tranexamic acid or aminocaproic acid are a therapeutic option. Given that antifibrinolytics increase the risk of circuit thrombosis (121), each case should undergo a benefit–risk assessment.

### 6.5 Hemolysis

Common triggers for device-associated hemolysis are high blood flow rates, very negative drainage pressures, suction events, and circuit thrombosis (98). When hemolysis occurs, actions to decrease contributing factors should be undertaken. These include a reduction of the blood flow and the minimization of oscillations in the drainage pressure (e.g., due to strong spontaneous breathing efforts or hypovolemia). Massive hemolysis may also be an indication of severe circuit thrombosis. In clinically relevant circuit thrombosis, additional signs are often detectable, including visible accumulation of fibrin or clots in the circuit, high transmembrane pressures, or a loss of gas exchange capability of the ECMO oxygenator. In this case, an exchange of the oxygenator or circuit will be necessary to stop the hemolysis (98).

### 6.6 Acquired Von Willebrand syndrome

Currently, there is not enough evidence to support treatment of ECMO-induced VWF deficiency with VWF-containing concentrates or desmopressin. Serious refractory bleeding complications may justify an attempt to restore HMW VWF multimers (50, 125). However, VWF supplementation may have limited effectiveness as long as the destructive stimulus is still in place. The only causal therapy for AVWS currently available is to remove device-induced shear stress (50). In the future, antibodies that target the interaction between ADAMTS13 and VWF may be a potential therapeutic option for patients who need ongoing extracorporeal support. These antibodies have been shown to stop the excessive degradation of high molecular weight VWF multimers in preclinical studies (126, 127).

### 6.7 Further measures to prevent bleeding complications

VV-ECMO patients appear to have a higher risk of intracranial hemorrhage compared to those on VA-ECMO, a phenomenon that remains not fully understood (128, 129). A drop in arterial carbon dioxide partial pressure (PaCO_2_) of more than 50% in 24 h is associated with an increased risk of neurological complications, including intracranial hemorrhage. Therefore, large decreases of PaCO_2_ should be avoided in the first 24 h after implementing VV-ECMO in patients with hypercapnia (130).

Emphasis should be placed on minimizing traumatic procedures in these patients. If invasive procedures such as chest tube placement or bronchoscopy are necessary, they should be performed with caution and by experienced personnel. Since bleeding of the airway is associated with a high mortality rate, excessive inline suctioning should be avoided.

## 7 Conclusion

A complex multifactorial coagulopathy characterized by imbalances between pro- and anticoagulant factors accompanies ECMO support. This coagulopathy is primarily mediated by shear-induced changes to primary hemostasis as well as contact activation due to exposure of the patient plasma to the foreign surfaces of the ECMO. In ECMO patients, disease-specific alterations of the coagulation system further complicate the balance to hemostasis. To avoid thrombotic complications anticoagulation is required during ECMO, but anticoagulation monitoring is challenging and requires specific drug monitoring. The evidence regarding hemostatic management during ECMO is still not satisfying. Nevertheless, concerning disease- and device-specific considerations, a personalized approach to hemostasis that balances the risk of thrombotic and bleeding complications should be aimed for. Bleeding due to shear-induced changes of the primary hemostasis will persist until termination of ECMO support despite optimal hemostatic management. This is why invasive procedures should be performed with extreme caution to keep the risk of bleeding complications as low as possible.
